# Structural Bases for the Regulation of CO Binding in the Archaeal Protoglobin from *Methanosarcina acetivorans*

**DOI:** 10.1371/journal.pone.0125959

**Published:** 2015-06-05

**Authors:** Lesley Tilleman, Stefania Abbruzzetti, Chiara Ciaccio, Giampiero De Sanctis, Marco Nardini, Alessandra Pesce, Filip Desmet, Luc Moens, Sabine Van Doorslaer, Stefano Bruno, Martino Bolognesi, Paolo Ascenzi, Massimo Coletta, Cristiano Viappiani, Sylvia Dewilde

**Affiliations:** 1 Department of Biomedical Sciences, University of Antwerp, Antwerp, Belgium; 2 Department of Physics and Earth Sciences, University of Parma, Parma, Italy; 3 Department of Clinical Sciences and Translational Medicine, University of Roma Tor Vergata, Roma, Italy; 4 Interuniversity Consortium for the Research on the Chemistry of Metals in Biological Systems, Bari, Italy; 5 Department of Biosciences, University of Milano, Milano, Italy; 6 Department of Physics, University of Genova, Genova, Italy; 7 Interdepartmental Laboratory of Electron Microscopy, University Roma Tre, Roma, Italy; 8 Department of Physics, University of Antwerp, Antwerp, Belgium; 9 Department of Pharmacy, University of Parma, Parma, Italy; The Chinese University of Hong Kong, CHINA

## Abstract

Studies of CO ligand binding revealed that two protein states with different ligand affinities exist in the protoglobin from *Methanosarcina acetivorans* (in *Ma*Pgb*, residue Cys(E20)101 was mutated to Ser). The switch between the two states occurs upon the ligation of *Ma*Pgb*. In this work, site-directed mutagenesis was used to explore the role of selected amino acids in ligand sensing and stabilization and in affecting the equilibrium between the “more reactive” and “less reactive” conformational states of *Ma*Pgb*. A combination of experimental data obtained from electronic and resonance Raman absorption spectra, CO ligand-binding kinetics, and X-ray crystallography was employed. Three amino acids were assigned a critical role: Trp(60)B9, Tyr(61)B10, and Phe(93)E11. Trp(60)B9 and Tyr(61)B10 are involved in ligand stabilization in the distal heme pocket; the strength of their interaction was reflected by the spectra of the CO-ligated *Ma*Pgb* and by the CO dissociation rate constants. In contrast, Phe(93)E11 is a key player in sensing the heme-bound ligand and promotes the rotation of the Trp(60)B9 side chain, thus favoring ligand stabilization. Although the structural bases of the fast CO binding rate constant of *Ma*Pgb* are still unclear, Trp(60)B9, Tyr(61)B10, and Phe(93)E11 play a role in regulating heme/ligand affinity.

## Introduction

*Methanosarcina acetivorans* (*M*. *acetivorans*) is an obligate anaerobic archaeon that was isolated from marine sediments where kelp decomposition, *e*.*g*., decomposition to methane, occurs [1]. The decomposing kelp produces CO that is presumed to be a substrate for *M*. *acetivorans* in nature. During CO-dependent growth, formate, CO_2_, and acetate are produced in addition to the expected methane, but strangely, no H_2_ is detected [2]. It was suggested that the pathway(s) for methane formation involves novel methyltransferases and a previously unreported mechanism for CO_2_ reduction [3]. Moreover, the formation of acetate from CO was proposed to be a remnant of a primitive energy-conservation cycle that drove and directed the early evolution of life on earth [4].

Because globins are heme proteins that can reversibly bind diatomic gaseous ligands, such as O_2_, CO and NO, globins might play undiscovered roles in the CO metabolism of *M*. *acetivorans*. An *in silico* search for globin genes in the fully sequenced genome revealed the presence of one gene coding for a protoglobin (Pgb) and this one was subsequently cloned, recombinantly expressed and crystallized. Pgbs are single-domain archaeal heme proteins that are related to the *N*-terminal domain of globin-coupled sensors (GCSs) [5].

**Fig 1:**
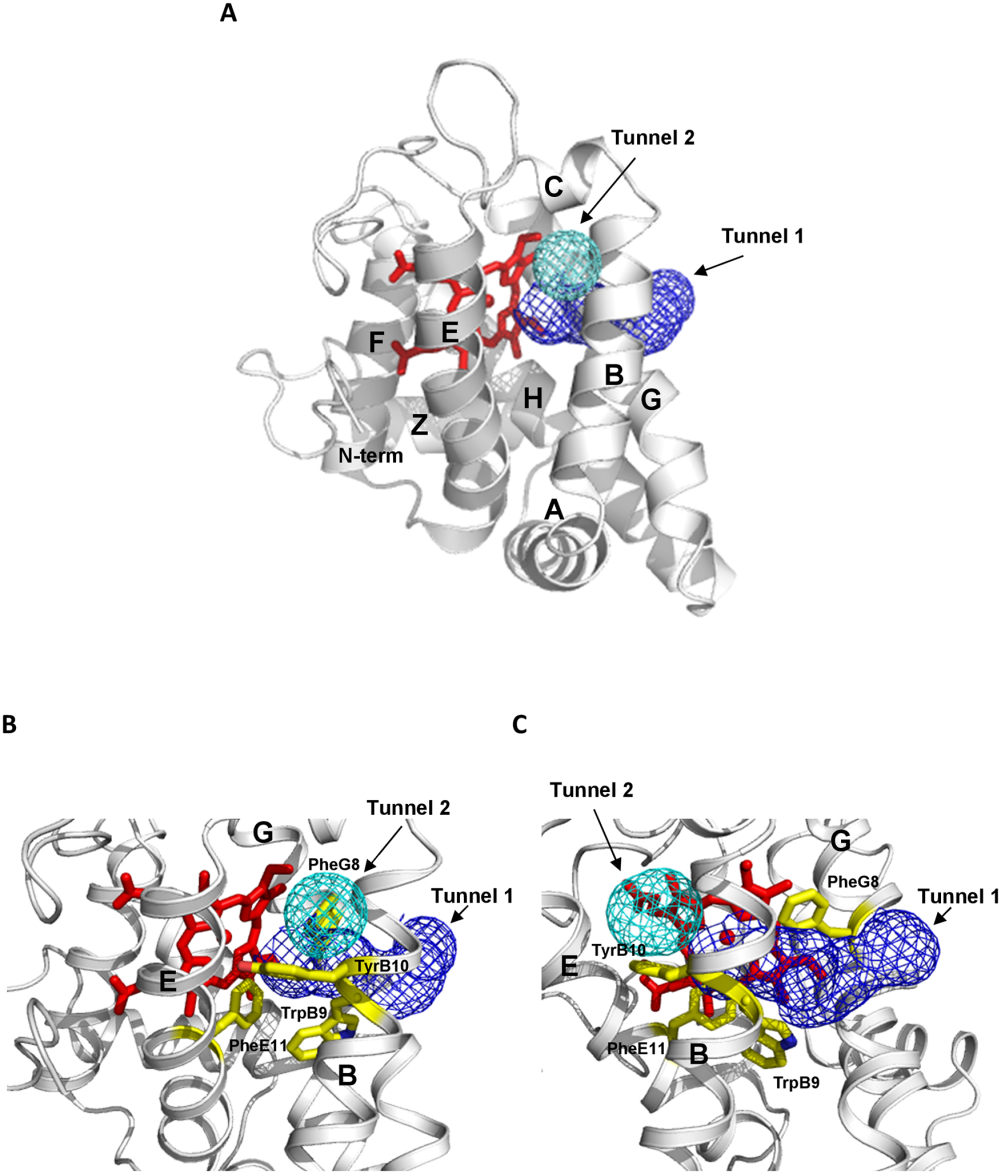
The *Ma*Pgb* tunnel system. **(A)** The figure highlights the secondary structure elements (gray; labels A through H) of the *Ma*Pgb* fold. The D helix of the canonical globin fold is absent in the *Ma*Pgb* fold, whereas an additional helix (named Z) precedes the A helix. The C-terminus of the protein is hidden in the back of the figure (after the H helix) and is therefore not labeled. The heme distal cavity is connected to the solvent region by tunnel 1 (blue mesh) and tunnel 2 (cyan mesh). The program Surfnet [28] was used to explore the protein matrix tunnels with a 1.4 Å radius probe. **(B)** Close-up view of the *Ma*Pgb* tunnel system in an orientation similar to that of panel A. Mutated residues (Phe(93)E11 in the distal pocket; Trp(60)B9 and Phe(145)G8 in tunnel 1; and Tyr(61)B10 in tunnel 2) are shown in stick representation (yellow) and labelled (for a better view, only the topological position of the residues is indicated). Relevant secondary structure elements are also indicated. **(C)** Figure similar to panel B but with an almost orthogonal orientation.

Several topological features of the crystal structure of *M*. *acetivorans* Pgb, whose residue Cys(E20)101 was mutated to Ser (*Ma*Pgb*), are unusual for a globin [6]. In addition to the typical 3-on-3 helical fold found in many globins, the heme group in MaPgb* is completely buried in the protein matrix by a 20-residue *N*-terminal loop followed by an extra Z-helix and some inter-helical loops with a previously characterized functional role [7]. Thus, the heme is only accessible through two orthogonal tunnels located at the B/G and B/E interfaces (named tunnel 1 and tunnel 2, respectively) (Fig 1). Through molecular dynamics (MD) computations, it was hypothesized that tunnel 2 (short and relatively polar) is always open, while the accessibility of tunnel 1 (long and apolar) is modulated by the dimerization and the ligation state of the protein [8]. In particular, it was stated that dimerization affects the spatial organization of helix G, which influences the shape of tunnel 1. Therefore, the orientation of the Phe(145)G8 side chain may regulate the accessibility of the ligand to the heme through tunnel 1, which can adopt open or closed conformations. It was also suggested that ligand sensing in the *Ma*Pgb* distal cavity could result from steric hindrance of the Phe(93)E11 side chain and of the heme-bound ligand [9]. Conformational changes occurring at Phe(93)E11 could alter the structural and dynamical behavior(s) of helices B and E, thus influencing the open/closed state of tunnel 1 [8]. CO ligand binding and dissociation studies demonstrated that the liganded protein adopts a tighter-binding conformation (*Ma*Pgb*^r^), and the unliganded protein adopts a weaker-binding conformation (*Ma*Pgb*^t^). Ligation favors the species with a high binding rate (and low dissociation rate). The equilibrium is shifted towards the species with low binding (and high dissociation) rates for the unliganded molecules. Biphasic CO binding kinetics was observed in flash-photolysis and stopped-flow experiments, showing that ferrous *Ma*Pgb*^r^ (*Ma*Pgb*^r^(II)) is characterized by a high CO association rate and low CO dissociation rate, whereas ferrous *Ma*Pgb*^t^ (*Ma*Pgb*^t^(II)) shows a low CO association rate and a high CO dissociation rate [10]. Although *Ma*Pgb* assembles into a homodimer in solution, this structure does not result in cooperative CO binding. Nevertheless, ligand binding induces specific conformational changes in both subunits [9].

The structural and dynamical details of the process(es) leading from the high-affinity liganded species to the low-affinity unliganded species are yet to be understood. Here, we present experimental evidence that provides insight into the role that critical amino acids of the distal heme pocket play in CO binding/sensing and in the adjustment of the conformational state.

## Material and Methods

### Recombinant expression and purification of MaPgb* mutants

Mutations were introduced in *Ma*Pgb using the QuikChange site-directed mutagenesis method (Stratagene), as described previously [11]. All site-directed mutants were obtained by starting from *Ma*Pgb*, which was produced for crystallization purposes only and bears the Cys(101)E20Ser substitution. Three types of mutants were designed: (*i*) the distal pocket mutants Phe(93)E11Ala and Phe(93)E11Tyr, (*ii*) the tunnel 1 mutants Trp(60)B9Ala and Phe(145)G8Trp, and (*iii*) the tunnel 2 mutant Tyr(61)B10Ala. The locations of the mutated residues in the three-dimensional structure of *Ma*Pgb* are shown in Fig 1, where the topological arrangement of the tunnels is also reported.

All recombinant proteins were expressed in *Escherichia coli* BL21(DE3)pLysS cells and collected as described previously [11]. The cells were resuspended in 50 mM Tris-HCl pH 8.0, 5.0 mM EDTA, 0.5 mM DTT and 1.0 mM phenylmethylsulphonyl fluoride. The bacterial cells were lysed, and recombinant proteins were purified from inclusion bodies as described previously [10].

### UV-Vis absorption spectroscopy

UV-Vis spectra of ferrous deoxygenated and carbonylated *Ma*Pgb* mutants (*Ma*Pgb*(II) and *Ma*Pgb*(II)-CO, respectively) were recorded in the 250–700 nm range using a Cary-5 UV-Vis-NIR spectrophotometer (Varian). Ferrous CO-bound and deoxygenated proteins were prepared as follows. The ferric *Ma*Pgb* (*Ma*Pgb*(III)) mutant stock solutions were diluted with 100 mM phosphate buffer at pH 7.0 to a final concentration of 50 μM, equilibrated in either 1.0 atm CO or N_2_ and anaerobically reduced by the addition of 10 μl of a saturated solution of sodium dithionite.

### Resonance Raman measurements of carbonylated *Ma*Pgb* mutants

Resonance Raman (RR) spectra of *Ma*Pgb*(II)-CO mutants were recorded using a Dilor XY-800 Raman scattering spectrometer consisting of a triple 800 mm spectrograph operating in low-dispersion mode and using a liquid nitrogen-cooled CCD detector as described previously [10]. The excitation source for all spectra was a mixed Ar/Kr laser (Spectra-Physics Beamlok 2060) operating at 413.1 nm. The spectra were recorded at room temperature. The *Ma*Pgb*(II)-CO solutions were stirred at 500 rpm to prevent local heating and photochemical decomposition in the laser beam. Five to ten spectra (120–240 s recording time each) were acquired to allow for the removal of cosmic ray spikes. These spikes were removed by eliminating the lowest and highest data points for each frequency value and averaging the remaining values. The laser power ranged from 0.5 to 100 mW. The protein concentration ranged between 45 and 78 μM (pH 8.5 at 20°C).

### CO-dissociation kinetics by stopped-flow experiments

The kinetics of CO dissociation from *Ma*Pgb*(II)-CO mutants was measured in 100 mM potassium phosphate buffer pH 7.0 at 20°C using a thermostatted stopped-flow apparatus (Applied Photophysics, Salisbury, UK) with a dead time of 1 ms [10].

The CO-dissociation rate constant was determined by mixing *Ma*Pgb*(II)-CO mutant solutions (10 μM) with 180 μM NO solution. The protein solutions were degassed, reduced with sodium dithionite (2.0 mM), exposed to CO and then extensively treated in nitrogen flux to remove excess CO. The NO solution was prepared by anaerobically dissolving the NO donor MAHMA NONOate (Sigma Aldrich) in a previously degassed buffer solution. The NO donor solution was left to equilibrate for approximately 30 min, a time much longer than that needed for the NO release, which occurs with a time constant of approximately 3 min. The exact concentration of NO was determined by titration with deoxygenated human hemoglobin (Hb) under anaerobic conditions. The displacement of CO by NO was monitored using the absorbance changes at 421 nm [10].

### Nanosecond laser flash photolysis

For laser photolysis, samples of *Ma*Pgb*(II)-CO mutants were prepared in a sealed 2 mm by 10 mm quartz cuvette. The ferric *Ma*Pgb* (*Ma*Pgb*(III)) mutant stock solutions were diluted with 100 mM phosphate buffer at pH 7.0 to a final concentration of 50 μM, equilibrated in either 1.0 atm or 0.1 atm CO and anaerobically reduced with sodium dithionite (2.0 mM). The oxidation state, the molar fraction of the protein-bound CO binding, and the heme concentration were determined spectrophotometrically.

Nanosecond flash-photolysis experiments and time-resolved difference absorbance spectra were measured as described [10,12] or with a laser photolysis system (Edinburgh Instruments LP920, Livingston, UK) using a frequency-doubled Q-switched Nd:YAG laser (Spectra Physics Quanta-ray, Newport, CA) at 532 nm.

### CO binding kinetics

The CO rebinding kinetics, which was measured using laser-photolysis experiments, was analyzed in the framework of Scheme 1 below [10]. After photodissociation of the bound state, which results in a mixture of MaPgb*^r^ *CO* and MaPgb*^t^ *CO*, CO can migrate from the primary docking site (MaPgb*^r^:*CO*)_1_/(MaPgb*^t^:*CO*)_1_ to a secondary docking site (MaPgb*^r^:*CO*)_2_/(MaPgb*^t^:*CO*)_2_ or can exit to the solvent resulting in MaPgb*^r^/MaPgb*^t^. Rebinding occurs through two distinct pathways involving either MaPgb*^t^ or MaPgb*^r^.

Different equilibrium constants connect MaPgb*^t^ with MaPgb*^r^ and MaPgb*^r^ *CO* with MaPgb*^r^ *CO*.

(MaPgb*r:CO)2↑kc↓k−cMaPgb*rCO←kg,r→hυ;kd,r(MaPgb*r:CO)1←kin,r→koutMaPgb*r+CO↑k−1↓k1↑k−2↓k2↑k−3↓k3MaPgb*tCO←kg,t→hυ;kd,t(MaPgb*t:CO)1←kin,t→koutMaPgb*t+CO↑kd↓k−d(MaPgb*t:CO)2 (1)

Global fitting of CO-rebinding traces at 0.1 and 1 atm CO was performed as described previously [10]. The differential equations were solved numerically within MATLAB using the ODE15s function. The rate constants, which were considered to be shared parameters between the progress curves collected at different CO pressures, were optimized using the package Minuit (CERN) to obtain a least-squares best fit to the experimental data [10,12–14]. Time-resolved difference spectra were analyzed by singular value decomposition (SVD) [12,15] using MATLAB (MathWorks, Inc., Natick, MA). Errors on parameters were determined by scanning across each parameter subspace in the vicinity of the minimum while the other parameters were held constant; the width of the resulting curve at a 10% increase in the fitness function was used as the error. Errors were further checked using the error matrix built during parameter optimization.

### Crystallization and data collection of the cyanide derivative of ferric Phe(93)E11Tyr and Phe(145)G8Trp *Ma*Pgb* mutants

The cyanide derivative of the ferric Phe(93)E11Tyr and Phe(145)G8Trp *Ma*Pgb* mutants (Phe(93)E11Tyr(III)-cyanide and Phe(145)G8Trp(III)-cyanide, respectively) was crystallized by vapor diffusion techniques (protein concentration ~2.6 mM) under conditions matching those for the ligand-free ferric protein[6]. The mutant protein solutions were equilibrated against a precipitant solution containing 10% (v/v) isopropanol, 20% (v/v) PEG 4000, 0.1 M Na-Hepes (pH 7.5), 0.02 M potassium ferricyanide, and 0.01 M KCN at 277 K. The crystals diffracted to 1.6 Å and 2.2 Å resolution for the Phe(93)E11Tyr(III)-cyanide and Phe(145)G8Trp(III)-cyanide mutants, respectively (at 100 K), using synchrotron radiation (ESRF, Grenoble, France); both crystals belong to the primitive monoclinic *P*2_1_ space group (S1 Table). All collected data were reduced and scaled using the programs MOSFLM and SCALA, respectively, [16,17] and were phased by molecular replacement methods using the program PHASER [18]; the structure of ferrous oxygenated *Ma*Pgb* (*Ma*Pgb*(II)-O_2_) was used as the starting model (PDB accession code 2VEB) [6]. The crystallographic refinement was performed using the program REFMAC [19], and the program COOT [20] was used for model building/inspection. The relevant refinement statistics are reported in S1 Table. The program Procheck [21] was used to assess the stereochemical quality of the protein structures. Atomic coordinates and structure factors for Phe(93)E11Tyr(III)-cyanide and Phe(145)G8Trp(III)-cyanide have been deposited using the PDB accession codes 3ZOL and 3ZOM, respectively.

## Results

### Electronic absorption spectroscopy

**Fig 2:**
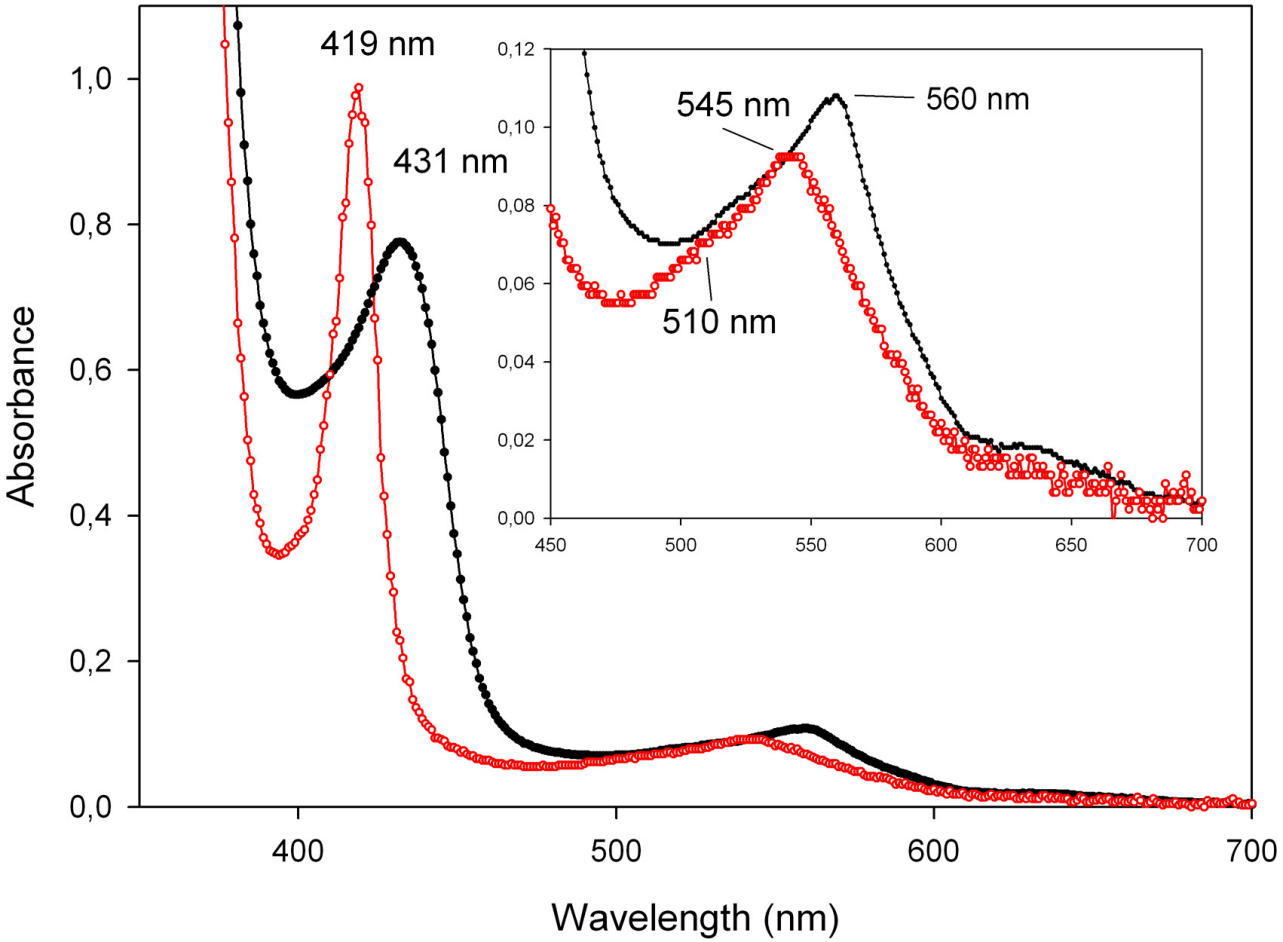
Ferrous deoxygenated (black line) and carbonylated (red line) derivatives of the Phe(93)E11Ala mutant as an example (other mutants show similar spectra). Spectra were obtained at pH 7.0 (100 mM potassium phosphate buffer) and 20.0°C.

All mutants have similar absorption spectra for their deoxy form and their carbonylated form, and these spectra resemble the absorption spectra of ferrous carbonylated *Ma*Pgb* and *Synechocystis* Hb (Fig 2). The deoxy *Ma*Pgb*(II) mutants exhibit a Q-band at 556 nm and a shoulder at 588 nm. The ferrous carbonylated forms display a single absorption maximum in the Q-band region at ~545 nm with a shoulder at 510 nm. The positions of the Soret peaks of all *Ma*Pgb*(II) and *Ma*Pgb*(II)-CO mutants range between 428 and 435 nm and between 418 and 423 nm, respectively.

### Resonance Raman spectra of *Ma*Pgb*(II), *Ma*Pgb*(II)-CO and related mutants

**Fig 3:**
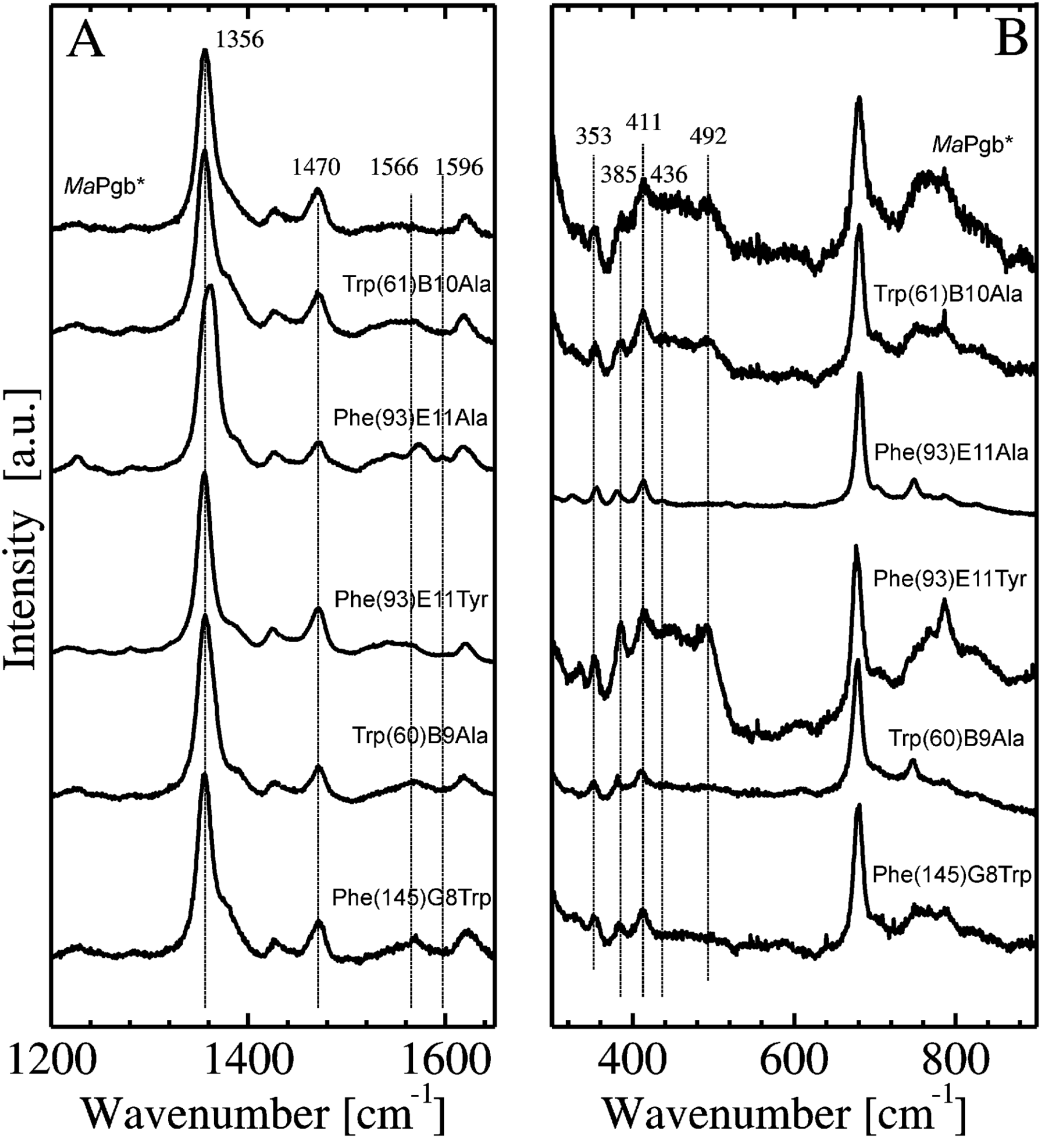
High-frequency (panel A) and low-frequency (panel B) regions of the RR spectra of *Ma*Pgb*(II) and related mutants. The RR spectra were recorded using a laser power of 50 mW.

**Fig 4:**
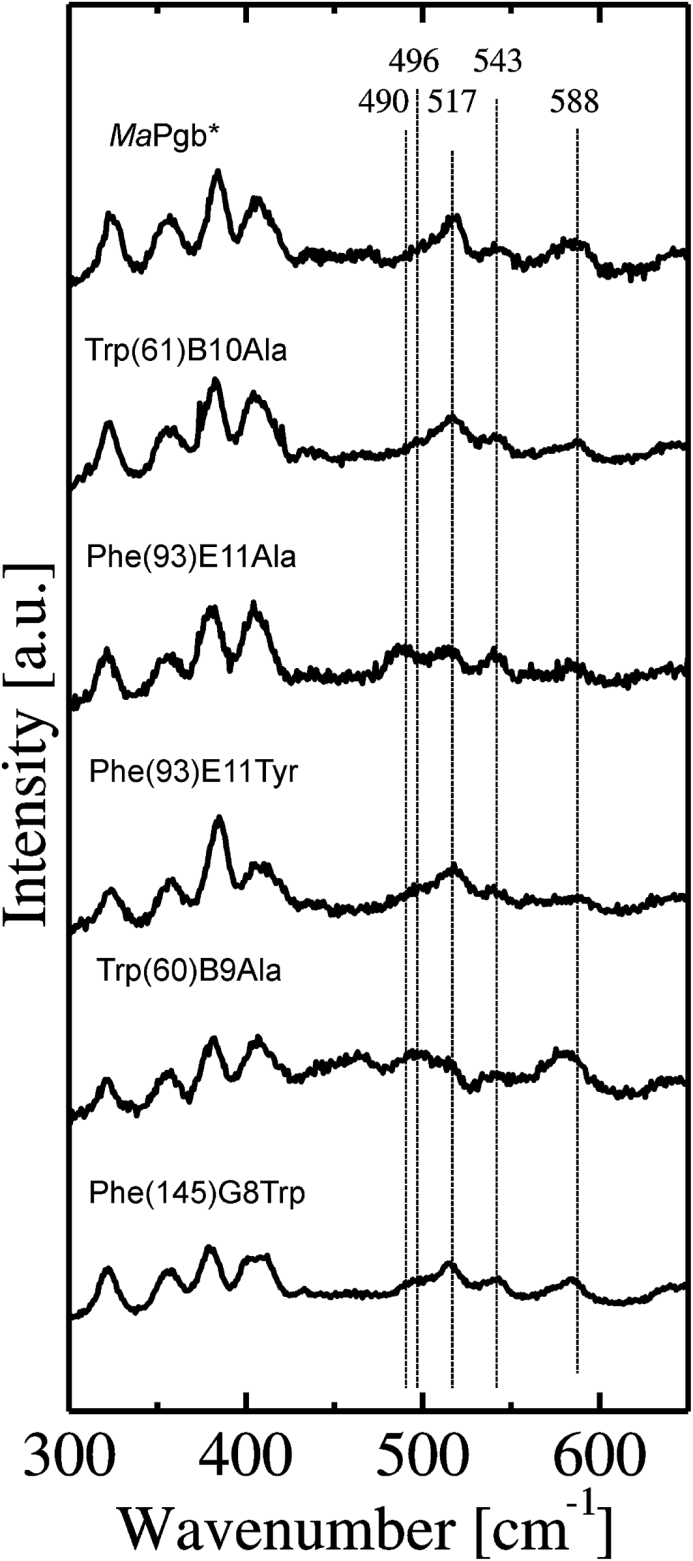
Low-frequency region of the RR spectra of *Ma*Pgb*(II)-CO and related mutants. The RR spectra were recorded using a laser power ranging between 0.5 and 2 mW.

The RR spectra of *Ma*Pgb*(II), *Ma*Pgb*(II)-CO and related mutants are shown in Figs 3 and 4, respectively. The marker bands in the high-frequency region for the ferrous deoxygenated mutants show very little variation (S2 Table). The oxidation marker band ν_4_ varies between 1354 and 1356 cm^-1^, while ν_3_ is located at ~1470 cm^-1^ for all mutants; these values are almost identical to those of *Ma*Pgb* ([10], Fig 3A). Only the position of the ν_2_ band, if at all visible, varies slightly between 1566 and 1571 cm^-1^ (Fig 3A). These values are typical of pentacoordinated, high-spin ferrous hemes (*S* = 2) [22]. The low-frequency region of the RR spectra of *Ma*Pgb*(II) mutants shows very little variation in the frequency of the propionate bending modes (Fig 3B), which are located between 383 and 385 cm^-1^, or in the frequency of the vinyl bending modes, which are located between 411 and 413 cm^-1^ (S2 Table).

Fig 4 shows the low-frequency regions of the RR spectra of *Ma*Pgb*(II)-CO mutants recorded at low laser power (≤2 mW). The frequency of the Fe-CO stretching mode (ν_Fe-CO_) depends on the strength of the interaction of the heme-bound CO with the amino acid residues present in the distal pocket [23]. The ν_Fe-CO_ frequencies in the 493–495 cm^-1^ range are associated with an open heme pocket, which is characterized by very few interactions between the heme-bound ligand and the polypeptide [24]. Frequencies in the range of 505–510 cm^-1^ are typical of mammalian myoglobins (Mbs) and Hbs containing a heme distal histidine at neutral pH [23]. The ν_Fe-CO_ frequencies in the 515–517 cm^-1^ range are associated with strong electrostatic interactions between the heme-bound CO and heme distal residues [25]. Higher frequencies of ~534 cm^-1^, as observed for barley Hb, are associated with the presence of positively polarized residues in the heme distal site; alternatively, these frequencies are associated, as in peroxidases, with an imidazolate character of the proximal histidine [25]. The value of the Fe-C-O bending frequency (δ (Fe-C-O)) is ~570 cm^-1^ [26]. A weak band at this position indicates an open heme pocket, whereas this band is strong in the case of strong interaction(s) between the heme-bound CO and the heme distal residues [24]. For barley Hb, the value of δ (Fe-C-O) is 586 cm^-1^ [23].

In our previous work [10], we reported that the heme distal side of *Ma*Pgb*(II)-CO is very heterogeneous. Inspection of Fig 4 immediately shows that the 490–600 cm^-1^ region of all *Ma*Pgb*(II)-CO mutants is also crowded with peaks indicative of a large heterogeneity of the heme distal site and that the fractions of the different conformations vary among these mutants (S3 Table). Tyr(61)B10Ala and Phe(145)G8Trp have intensities and positions of the CO modes that are comparable to those of *Ma*Pgb*. In fact, these mutants only exhibit weak bands at ν_Fe-CO_ < 500 cm^-1^, indicating a predominant presence of the closed conformation, and a maximum at ~517 cm^-1^, indicating strong interactions between CO and amino acid residues in the heme distal pocket. Additionally, these mutants share a ν_Fe-CO_ frequency mode at 543 cm^-1^, along with a broad δ (Fe-C-O) band near 588 cm^-1^ (Fig 4). The ν_Fe-CO_ modes of *Ma*Pgb* Phe(93)E11Tyr(II)-CO are very similar to those of *Ma*Pgb*(II)-CO, but both the δ (Fe-C-O) modes and the corresponding 543 cm^-1^ modes are less intense (Fig 4). Phe(93)E11Ala(II)-CO has the most intense ν_Fe-CO_ band at 490 cm^-1^, indicating a shift of the equilibrium towards the open carbonylated from. For Trp(60)B9Ala(II)-CO, the intermediately closed form with ν_Fe-CO_ at approximately 500 cm^-1^ dominates the spectrum, and the closed haem pocket conformation is decreased if compared to the spectra of *Ma*Pgb*(II)-CO.

### CO dissociation kinetics measured using stopped flow experiments

Similarly to the *Ma*Pgb*(II)-CO dissociation [10], the CO dissociation from most of the investigated mutants is biphasic. The time constants and amplitudes are reported in Table 1. The only remarkable change is observed in the amplitudes of the CO dissociation reaction of Phe(93)E11Ala(II)-CO. In fact, the fast dissociation phase shows a larger amplitude than that of *Ma*Pgb*(II)-CO; this finding is in agreement with the larger contribution of the open ferrous carbonylated form (Fig 4). In contrast, the CO dissociation from Trp(60)B9Ala(II)-CO follows a nearly mono-exponential process.

**Table 1:** Values of the microscopic rate constants for CO rebinding kinetics after laser photolysis at 20°C.

	*Ma*Pgb*	Trp(60)B9Ala	Phe(93)E11Tyr	Phe(93)E11Ala	Tyr(61)B10Ala	Phe(145)G8Trp
*k*_*1*_ (10^5^ s^-1^)	1.3±0.9	1.4±0.8	1.4±0.9	1.35±0.7	1.35±0.8	1.35±0.9
*k*_*-1*_ (10^5^ s^-1^)	5±2	2±2	3±3	0.8±0.9	4±4	5±6
*k*_*3*_ (10^4^ s^-1^)	6±2	0.5±0.1	15±4	4±1	0.6±0.2	4±1
*k*_*-3*_ (10^4^ s^-1^)	2.0±0.3	0.9±0.5	2±1	1.7±0.8	0.6±0.3	1.7±0.8
*k*_*in*,*r*_ (10^7^ M^-1^ s^-1^)	7.8±0.5	40±3	38±2	37±2	70±4	35±2
*k*_*in*,*t*_ (10^7^ M^-1^ s^-1^)	3.0±0.1	3.0±0.1	3.0±0.1	1.0±0.1	0.3±0.3	3.0±0.1
*k*_*out*_ (10^8^ s^-1^)	1.5±0.3	2.0±0.3	1.4±0.1	0.7±0.3	4.5±0.3	0.9±0.2
*k*_*g*,*r*_ (10^7^ s^-1^)	5±1	5±3	2±4	5±4	6±4	4±3
*k*_*g*,*t*_ (10^6^ s^-1^)	6.0±0.7	5±3	6±3	6±1	6.0±0.3	5±3
*k*_*c*_ (10^7^ s^-1^)	1.0±0.3	5±1	5±1	1.0±0.3	1.0±0.3	0.7±0.2
*k*_*-c*_ (10^7^ s^-1^)	1.0±0.2	5±5	1±1	1±1	1±1	3±3
*k*_*d*_ (10^7^ s^-1^)	1.0±0.1	5±5	5±5	1±1	1±1	0.7±0.7
*k*_*-d*_ (10^7^ s^-1^)	1.0±0.2	5±3	1.0±0.6	1.0±0.6	1.0±0.6	3±2
*k*_*ON*,*r*_ (×10^7^ M^-1^ s^-1^)	2.1±0.4	8±1	16±1	6±1	7.6±0.9	1.0±0.8
*k*_*ON*,*t*_ (×10^6^ M^-1^ s^-1^)	1.1±0.4	0.7±0.6	0.8±0.7	1.3±0.7	0.4±0.3	1.5±0.9
*k*_*OFF*,*2*_ (s)	0.032± 0.006 (67%)	0.020±0.006 (100%)	0.020±0.005 (24%)	0.21±0.03 (66%)	0.050±0.005 (68%)	0.100±0.012 (67%)
*k*_*OFF*,*1*_ (s)	0.081±0.014 (33%)	-	0.24±0.03 (76%)	0.40±0.06 (34%)	0.23±0.04 (32%)	0.50±0.09 (33%)

The rates were determined by fitting the experimental rebinding curves to reaction Scheme 1. The table also shows the second-order rate constants of CO binding that were calculated from the microscopic rate constants and shows the amplitudes and apparent rate constants of CO dissociation.

The rates *k*_*2*_ and *k*_*-2*_ are expected to be the same order of magnitude as the rates *k*_*3*_ and *k*_*-3*_. Because the analysis provides unreliable values for *k*_*2*_ and *k*_*-2*_, these parameters were set equal to *k*_*3*_ and *k*_*-3*_, respectively; the latter two parameters are determined with much better precision.

As previously observed for the WT protein, the rates *k*_*c*_, *k*_*-c*_, *k*_*d*_, and *k*_*-d*_ are also difficult to estimate for these mutants given the small amplitude of the corresponding processes.

The rate equations for CO binding have poor sensitivity to *k*_*d*,*r*_ and *k*_*d*,*t*_, which are held constant to the values determined by the following equation: *k*_*OFF*,*1/2*_ = *k*_*d*,*r/t*_ × *k*_*out*_ /(*k*_*out*_ + *k*_*g*,*r/t*_), where *k*_*OFF*,*1/2*_ is the dissociation rate constant for CO determined by mixing CO*Ma*Pgb* with NO.

### CO rebinding kinetics to *Ma*Pgb*(II) and related mutants after flash photolysis

**Fig 5:**
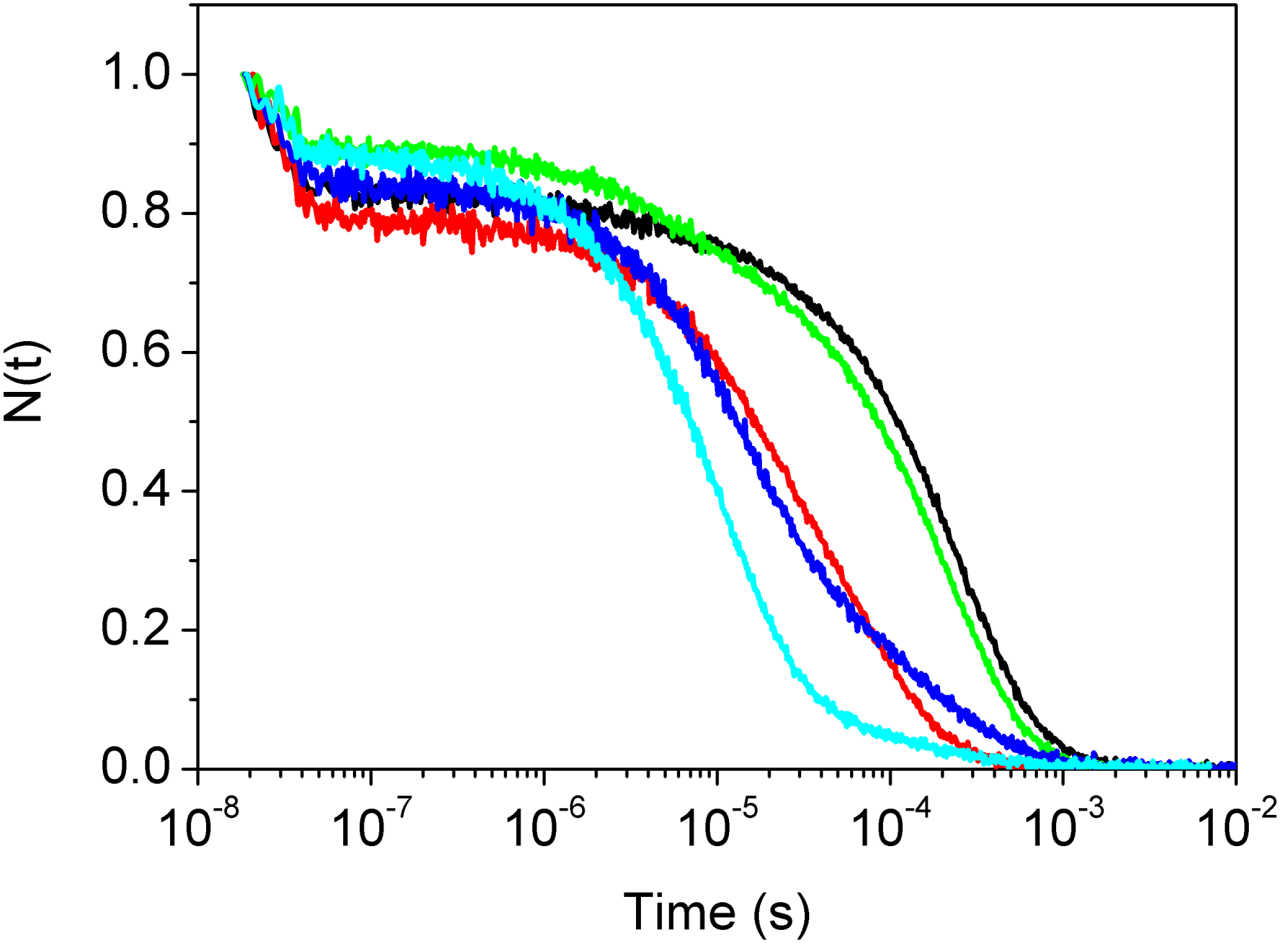
Kinetics of CO rebinding to *Ma*Pgb*(II) (black), Phe(93)E11Ala(II) (red), Phe(93)E11Tyr(II) (green), Trp(60)B9Ala(II) (blue), and Tyr(61)B10Ala(II) (cyan). CO = 1.0 atm. T = 20.0°C and pH 7.0.

Fig 5 compares the observed rebinding kinetics of *Ma*Pgb*(II) and its mutants. It is quite evident that the selected mutations only exert a minor effect on the geminate phase. In contrast, the influence on the shape of the bimolecular phase is remarkably different. Some mutants, such as Phe(93)E11Tyr(II), display rebinding kinetics that is very similar to that observed for *Ma*Pgb*(II), suggesting that the rate constants for ligand binding and migration and for structural relaxation are essentially unchanged.

However, Phe(93)E11Ala(II) Tyr(61)B10Ala(II), and Trp(60)B9Ala(II) display bimolecular phases with strongly altered apparent rate constants and relative amplitudes. This finding suggests that these mutations have a substantial effect on CO rebinding and on the equilibrium between the *Ma*Pgb*^r^(II) and *Ma*Pgb*^t^(II) conformations of the CO-bound species and during CO rebinding after very fast laser photodissociation.

**Fig 6:**
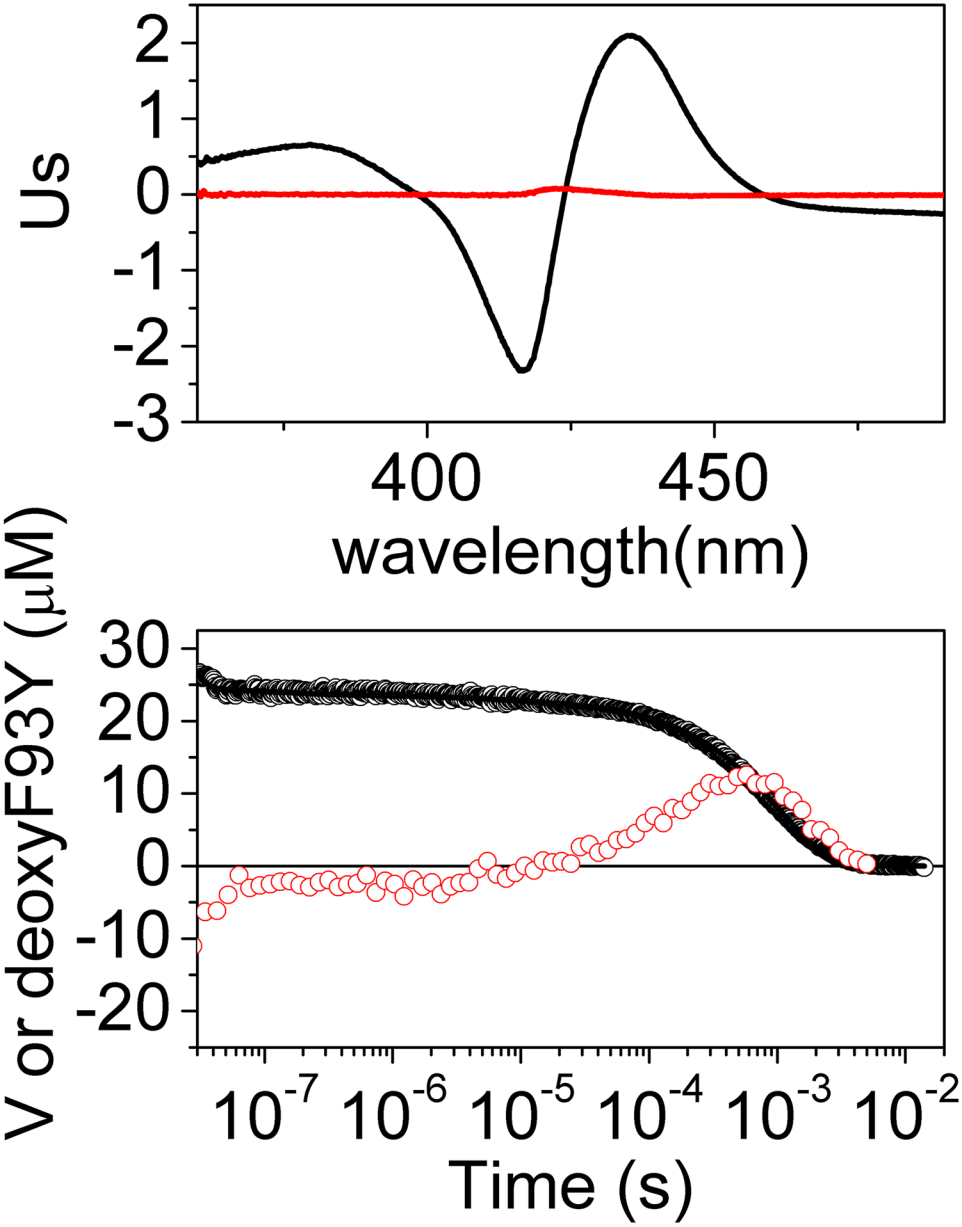
Meaningful spectral components (panel A) and amplitudes (panel B) as determined using the SVD analysis of the time-resolved spectra collected after photolysis of the *Ma*Pgb* Phe(93)E11Tyr(II)-CO mutant. The first component (black open circles, S_1_ = 27, corr_1_ = 0.99) matches the rebinding kinetics measured at 436 nm (black solid curve), whereas the second component (red open circles, S_2_ = 0.6, corr_2_ = 0.99) shows the formation and decay of a reaction intermediate, as observed for *Ma*Pgb*(II)-CO.

The CO rebinding kinetics of the mutants has been analyzed and compared to that of *Ma*Pgb*(II). When Phe(93)E11Tyr(II) and Tyr(61)B10Ala(II) are considered, the time-resolved absorbance spectra clearly show a spectral change with a shape and time profile that resembles the profile of *Ma*Pgb*(II). Fig 6 and S1 Fig show the two meaningful spectral components and their amplitudes as determined using the SVD analysis of Phe(93)E11Tyr(II) and Tyr(61)B10Ala(II), respectively.

**Fig 7:**
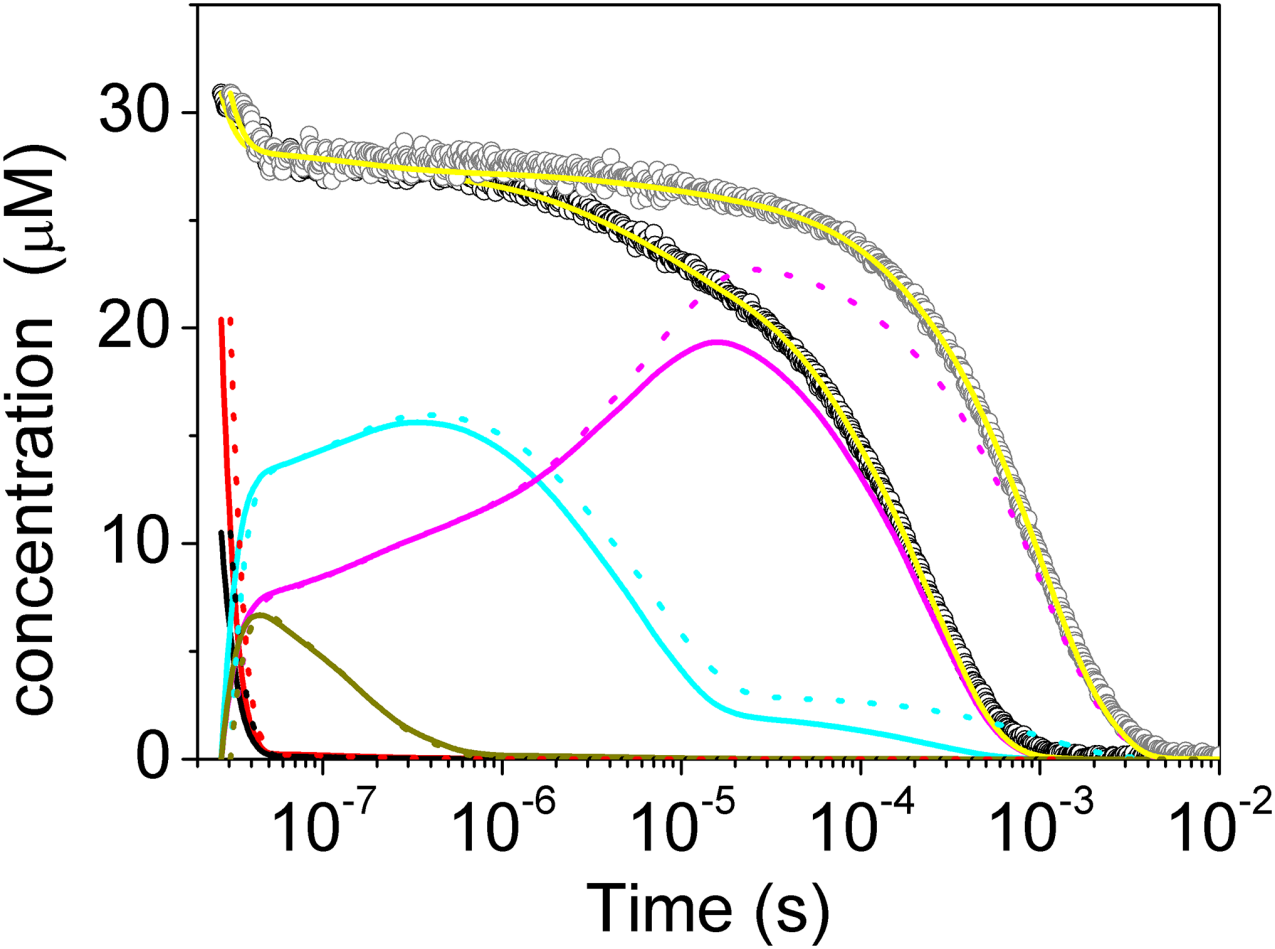
Kinetics of CO rebinding to the *Ma*Pgb* Phe(93)E11Tyr at 1 atm CO (black open circles) and 0.1 atm CO (gray open circles). T = 20°C. Yellow solid and dotted lines are the results of the fits to the experimental curves using the kinetic model developed for *Ma*Pgb* [9] and reported as Scheme 1. Reaction intermediates for 1 atm CO (solid lines) and 0.1 atm CO (dotted lines) are also reported.

The analysis of the kinetics of CO rebinding to Phe(93)E11Tyr(II) according to Scheme 1 is shown in Fig 7. As in the case of *Ma*Pgb*(II), the switching from *Ma*Pgb*^r^(II) (cyan) to *Ma*Pgb*^t^(II) (magenta) is quite evident, in agreement with the spectral relaxation (Fig 5). The extent of the conformational change is larger at low CO pressure because the rebinding rate becomes slower and thus provides more time for the formation of the slow-reacting form.

The kinetics of CO rebinding to Phe(145)G8Trp(II) is also very similar to that of *Ma*Pgb*(II). S2 Fig shows representative fitting curves that demonstrate the existence of conformational switching with an extent and kinetics that are not substantially different from those observed for *Ma*Pgb*(II). The fitting parameters are reported in Table 1.

The kinetics of CO rebinding to Phe(93)E11Ala(II) and Trp(60)B9Ala(II) is quite different from those of the other mutants (see Fig 5). The geminate rebinding is similar to that observed for *Ma*Pgb*(II). The bimolecular CO rebinding to Phe(93)E11Ala(II), which is easily identified as the portion of the rebinding kinetics that is affected by the CO concentration, can be well described by a double exponential decay with lifetimes (amplitudes) of 14 μs (37%) and 84 μs (63%) at 1 atm CO. The lifetimes increase to 106 μs (39%) and 522 μs (61%) at 0.1 atm CO, with very little change in the amplitudes of the two transients. This result suggests that no conformational change occurs upon photolysis before CO rebinding. Consistently, only one meaningful spectral component was found using the SVD analysis of the time-resolved spectra; this component is coincident with the carboxy-*minus*-deoxy absorption spectrum (data not shown).

The RR data support the absence of a conformational switch (see Fig 4) and indicate the following: a prevailing open conformation of Phe(93)E11Ala(II)-CO, as judged from the intense ν_Fe-CO_ band at 490 cm^-1^, and an intermediately closed form for Trp(60)B9Ala(II)-CO, with a ν_Fe-CO_ band at approximately 500 cm^-1^.

**Fig 8:**
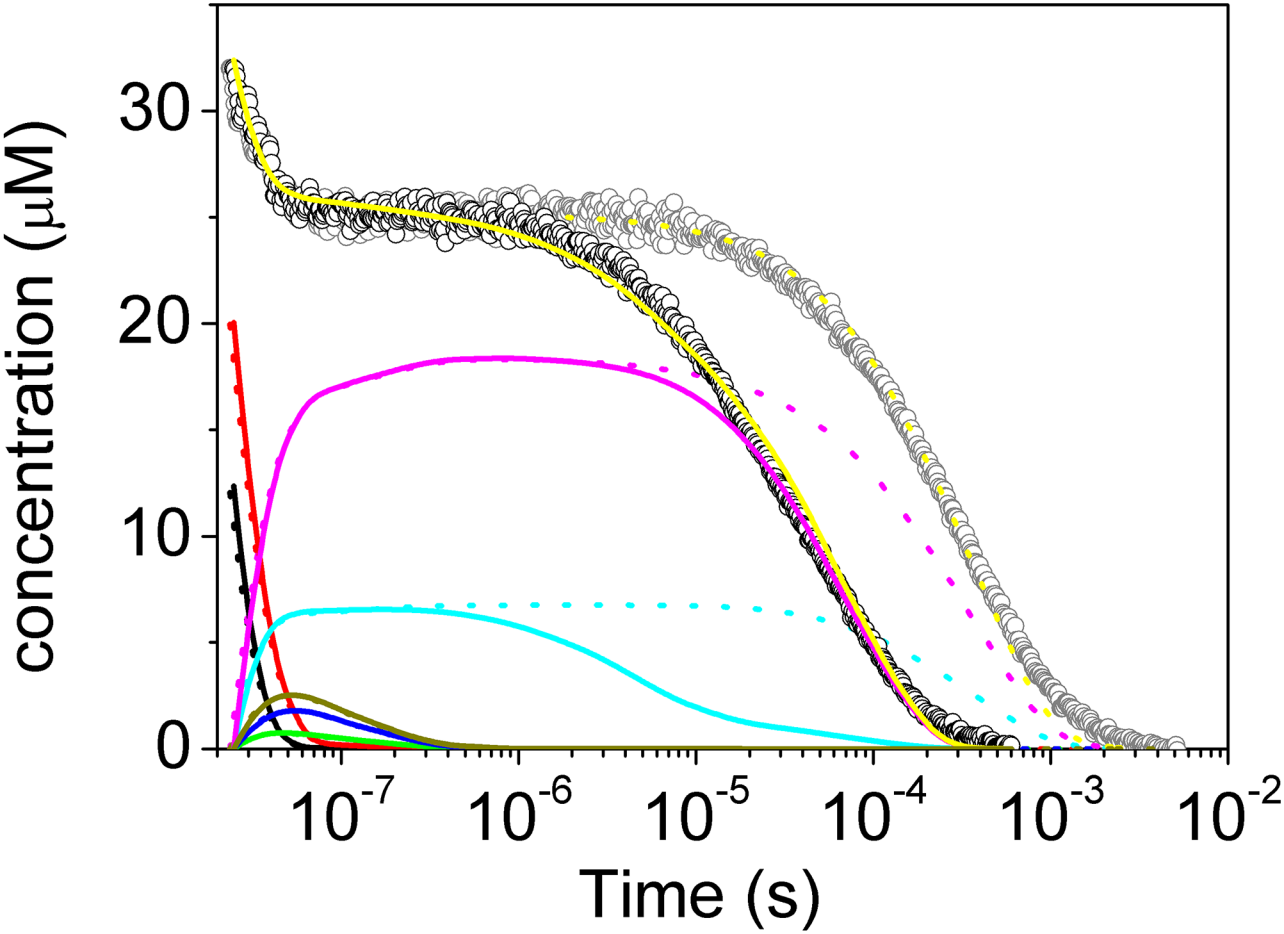
Kinetics of CO rebinding to Phe(93)E11Ala(II) at 1.0 atm CO (black open circles) and 0.1 atm CO (gray open circles). T = 20.0°C and pH 7.0. Yellow solid and dotted lines are the results of the fits to the experimental curves using the kinetic model developed for *Ma*Pgb* [9] and reported as Scheme 1. Reaction intermediates obtained at 1 atm CO (solid lines) and 0.1 atm CO (dotted lines) are also reported.

Analyses of the kinetics of CO rebinding to Phe(93)E11Ala(II) and Trp(60)B9Ala(II) are reported in Fig 8 and S3 Fig, respectively. The calculated microscopic rate constants are reported in Table 1. As shown in Fig 8, no interconversion occurs between *Ma*Pgb*^r^(II) and *Ma*Pgb*^t^(II). The relative concentration of each of these species, once formed, is independent of the time after photolysis and decays with CO rebinding.

### Crystal structures of the Phe(93)E11Tyr(III)-cyanide and Phe(145)G8Trp(III)-cyanide mutants

Attempts to crystallize the ferrous form of *Ma*Pgb* (WT or mutants) in complex with CO did not result in any crystal suitable for X-ray data collection. However, the crystallization of ferric *Ma*Pgb* (WT or mutants) in complex with cyanide provided reproducible and well-diffracting crystals. Therefore, we used the *Ma*Pgb*(III)-cyanide complex as a model for the structural interpretation of the diatomic ligand-binding properties of *Ma*Pgb*. Here, we report the crystal structures of the Phe(93)E11Tyr(III)-cyanide and Phe(145)G8Trp(III)-cyanide complexes (data statistics in S1 Table). In both cases, the mutation does not alter the global structure, as indicated by the rms deviation values between 0.15 and 0.45 Å (calculated for 190 C_α_ atom pairs) for the structure of the cyanide derivative of ferric *Ma*Pgb* (*Ma*Pgb*(III)-cyanide) [9] and the structure of ferrous oxygenated *Ma*Pgb* (*Ma*Pgb*(II)-O_2_) [6]. Similar considerations apply to the conserved dimeric assembly; this assembly is based on the following: a four-helix bundle built by the G- and H-helices, the H’-helix, partly the Z-helix, and the BC and FG hinges [6]. Both ferric *Ma*Pgb* mutants have been crystallized in the presence of cyanide, which is clearly visible in the electron density of the Phe(93)E11Tyr mutant as the heme-Fe(III)-coordinated ligand but not for the Phe(145)G8Trp mutant, where the heme-Fe(III) atom appears to be free of a distal ligand.

**Fig 9:**
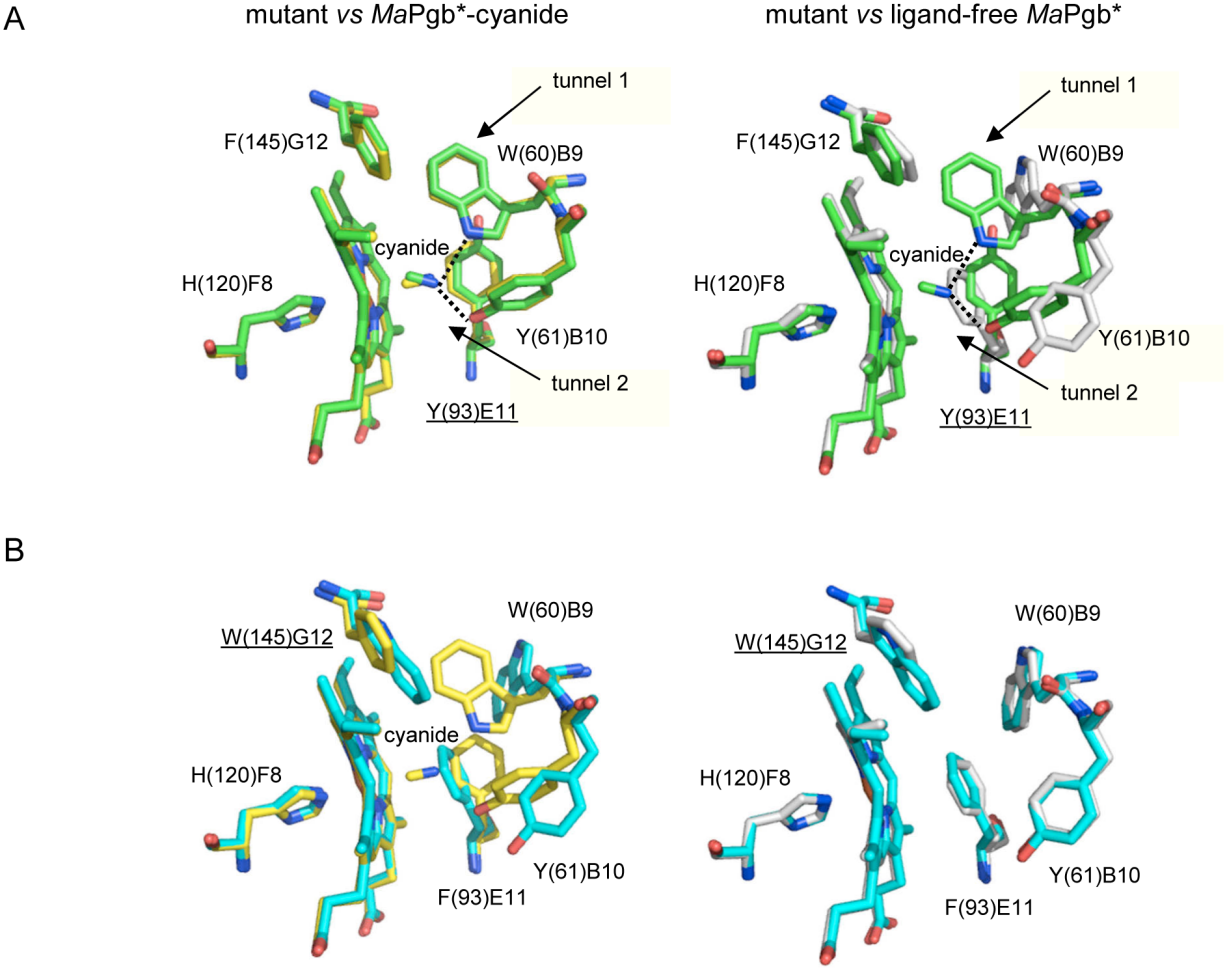
Structures of the heme distal site of *Ma*Pgb*(III)-cyanide mutants. Residues lining the heme distal pocket are indicated (one letter code) and shown in green for the Phe(93)E11Tyr mutant (panel A) and in cyan for the Phe(145)G12Trp mutant (panel B). The proximal His(120)F8 residue is also shown. The cyanide-bound and ligand-free *Ma*Pgb*(III) structures (taken as references) are shown in yellow and grey colors, respectively. The mutated residues have been underlined. Hydrogen bonds are shown as dashed lines. In panel A, the location of tunnel 1 and tunnel 2 is schematically indicated.

In Phe(93)E11Tyr(III)-cyanide, the ligand is bound to the heme-Fe(III) atom with a coordination bond of 2.41 Å and a Fe-C-N angle of 144.7° (2.50 Å and 134.0° in the second dimer subunit). The coordination bond length for cyanide is slightly longer than expected, likely due to partial photoreduction of the heme-Fe(III). Two hydrogen bonds stabilize the heme-Fe(III)-bound cyanide, its N atom being linked to the Tyr(61)B10 OH (2.63 Å and 2.57 Å in the two subunits), and to the Trp(60)B9 N _Ɛ2_ atom (2.92 and 3.11 Å in the two subunits) (Fig 9A). The distal site structure and the cyanide-binding mode observed for the Phe(93)E11Tyr(III)-cyanide complex is closely reminiscent of the ligand-binding mode reported for *Ma*Pgb*(III)-cyanide [9]. The Tyr(93)E11 side chain of the mutated residue matches the position of the *Ma*Pgb*(III)-cyanide Phe(93)E11 residue, with only a small rotation (approximately 5–8°) away from the heme (Fig 9A). Thus, in both *Ma*Pgb*(III)-cyanide and Phe(93)E11Tyr(III)-cyanide, the aromatic side chain at topological position E11 rotates approximately 90° relative to the position in *Ma*Pgb*(III) to provide room for cyanide to bind to Trp(60)B9. As a result of the new Trp(60)B9 side chain location, tunnel 1 is closed in both *Ma*Pgb*(III)-cyanide and Phe(93)E11Tyr(III)-cyanide (Fig 9A).

The Phe(145)G8Trp mutation results in a composite rearrangement of the heme distal site cavity, with side-chain crowding that prevents cyanide binding. In the mutant, the side-chain locations of Phe(93)E11, Trp(60)B9, and Tyr(61)B10 match those found in the native ligand-free *Ma*Pgb*(III) structure [6] (Fig 9B). As a result, tunnel 1 is open because the mutated Trp(145)G8 side chain is not sufficient to hinder tunnel 1. Interestingly, previous EPR experiments indicated that the cyanide derivative of the ferric *Ma*Pgb* mutant Phe(145)G8Trp can occur but always as a mixture of high-spin Fe(III) and cyanide-ligated species (EPR can only detect the ferric oxidation states of the protein) [27].

The crystal structures of Trp(60)B9Ala(III)-cyanide and Tyr(61)B10Ala(III)-cyanide have been reported elsewhere [9]. In Trp(60)B9Ala(III)-cyanide, only the hydrogen bond to the Tyr(61)B10 OH group is present (2.74 Å and 2.59 Å, depending on the subunit). As a result, the heme-Fe(III)-bound cyanide is more evidently oriented toward the entrance of tunnel 2, where Tyr(61)B10 is located. Furthermore, in the Trp(60)B9Ala mutation, tunnel 1 always remains in its open form (S4 Fig). Interestingly, the location of the Phe(93)E11 side chain in this mutant matches that found in *Ma*Pgb*(II)-cyanide, suggesting that the heme-Fe(III)-bound cyanide induces the rotation of Phe(93)E11 required for the insertion of the Trp(60)B9 side chain into the heme distal site.

The Tyr(61)B10Ala mutation dramatically alters the accessibility of the heme distal site because the mutation increases the average diameter of tunnel 2 by more than 1.5 Å (with ~6.0 Å as the smallest distance between the Ala(61)B10 C_β_ atom and the surrounding residues). In the Tyr(61)B10Ala mutant structure, the heme-Fe(III)-bound cyanide is stabilized by the interaction with the Trp(60)B9 N _Ɛ2_ atom (2.90 Å and 2.85 Å in the two subunits). As a result, the orientation of the heme-Fe(III)-bound ligand is almost perpendicular to the heme plane (Fe-C-N angle of 179.0° and 176.7°) with Fe(III)-coordination bonds of 2.05 Å and 2.08 Å, respectively. Tunnel 1 is closed by the Trp(60)B9 side chain, and the positions of all residues lining the cavity at the heme distal site match the positions found in *Ma*Pgb*(III)-cyanide (S4 Fig).

## Discussion

The binding of diatomic ligands to heme proteins has long been known to lead to tertiary structural rearrangements, resulting in changes in reactivity. We believe that protoglobin is an excellent model system to study the molecular mechanisms and control of heme reactivity.

The available experimental data on the CO binding kinetics of *Ma*Pgb*(II) show that an equilibrium exists between two tertiary conformations of the liganded Pgb: *Ma*Pgb*^r^(II)-CO and *Ma*Pgb*^t^(II)-CO (with an equilibrium constant *K*_1_ = *k*_1_/*k*_-1_ 0.3; see Table 2); this equilibrium favors *Ma*Pgb*^r^(II)-CO over *Ma*Pgb*^t^(II)-CO [10].

**Table 2:** Values of the equilibrium binding constants derived from the analysis of CO rebinding curves.

	*K*_*1*_ = *k*_*1*_/*k*_*-1*_	*K*_*3*_ = *k*_*3*_/*k*_*-3*_
*Ma*Pgb*	0.3±0.2	3.0±0.4
*Ma*Pgb* Trp(B9)60Ala	0.7±1.1	0.5±0.4
*Ma*Pgb* Phe(E11)93Tyr	0.5±0.7	7.5±5.8
*Ma*Pgb* Phe(E11)93Ala	2±2	2.4±1.7
*Ma*Pgb* Tyr(B10)61Ala	0.3±0.5	1.0±0.8
*Ma*Pgb* Phe(G8)145Trp	0.25±0.5	2.4±1.7

The crystal structures of *Ma*Pgb*(III)-cyanide and related mutants provide a general structural model for understanding the roles of the residues in the heme distal-pocket involved in ligand stabilization, and this knowledge may be applied to the MaPgb*(II)-CO complex. The involvement of more than one amino acid residue in the hydrogen-bond interactions with the heme-bound ligand, namely Trp(60)B9 and Tyr(61)B10, can explain the observed heterogeneity in the spectroscopic data. The heterogeneous ν_Fe-CO_ frequencies in the 490–517 cm^-1^ range for the ferrous carbonylated *Ma*Pgb* mutants (Fig 4) can be associated with the existence of different degrees of polar interactions in the heme distal pocket; these interactions range from negligible (490 cm^-1^, open distal pocket) to weak (496 cm^-1^) and to strong (517 cm^-1^, closed distal pocket). The strongest hydrogen-bond interaction with the bound CO appears to be provided by the Trp(60)B9 side chain. Mutating Trp(60)B9 to Ala removes the interaction with the heme-bound ligand and induces a shift of the equilibrium to the more open conformation of the distal site (517 cm^-1^, Fig 4). In contrast, the Tyr(61)B10Ala mutation has small effects on the ν_Fe-CO_ frequency, suggesting only minor involvement of this residue in the stabilization of the heme-bound CO and a possible contribution of this residue to weak (496 cm^-1^) polar interactions. In this regard, CO appears to behave slightly differently from cyanide, whose strongest H-bond is provided by Tyr(61)B10 [9].

The CO dissociation kinetics (Table 1) confirms the above picture. The CO dissociation kinetics from Tyr(61)B10Ala(II)-CO is essentially identical to that observed for *Ma*Pgb*(II)-CO; the equilibrium constant *K*_1_ = *k*_1_/*k*_-1_ is consistently approximately 0.3. In contrast, when the CO dissociation from Trp(60)B9Ala(II)-CO is considered, the observed dissociation kinetics is almost monophasic and faster than that for *Ma*Pgb*(II)-CO and can be correlated with the loss of the strongest ligand-stabilizing interaction. Upon CO binding, the side chain of the Ala(60)B9 of the Trp(60)B9Ala mutant is clearly not capable of transducing the rotation of the Phe(93)E11 side chain into a stabilizing interaction with the bound ligand, nor is it able to close channel 1, which remains in an open conformation. This result confirms the hypothesis that Trp(60)B9 is responsible for the closed conformation of tunnel 1 in the heme distal site and for the high-affinity species.

The role of Phe(93)E11 in sensing the presence of the heme-bound ligand and triggering the structural response can be discerned by comparing the crystal structures of the liganded and unliganded *Ma*Pgb*(III), as in S4 Fig [9]. The mutation of Phe(93)E11 to Ala, a much smaller side chain, is accompanied by strong effects on the CO dissociation kinetics. The weight of the slower dissociation phase becomes much smaller for this mutant, indicating that the stabilization of the heme-bound ligand is much less effective than that for *Ma*Pgb*(II)-CO. In keeping with this observation, the 517 cm^-1^ band in the RR spectrum of Phe(93)E11Ala(II)-CO is almost absent. The above properties are reflected in the value of the equilibrium constant *K*_1_ = *k*_1_/*k*_-1_ that becomes ≈ 1.7 (Table 2). In addition, no switching between the r and t states is observed after photolysis, confirming that the structural rearrangement that is operative in *Ma*Pgb* is absent in the Phe(93)E11Ala mutant. Not surprisingly, the equilibrium constant *K*_3_ = *k*_*3*_/*k*_*-3*_ = 2.1 is similar to *K*_1_ = *k*_1_/*k*_-1_ = 1.7 (Table 2).

The consequences of the Phe(93)E11Tyr mutation are quite small, likely because of the comparable sizes of the Phe and Tyr side chains. The RR data on the Phe(93)E11Tyr(II)-CO mutant show ν_Fe-CO_ modes that are very similar to those of *Ma*Pgb*(II)-CO and point to a similar environment for the heme-bound CO. The similar sizes of the Phe and Tyr side chains likely allow for similar sensing of the heme-bound CO, resulting in the same type of pressure to undergo the conformational rearrangement of Trp(60)B9 (Fig 9A). The values of *K*_3_ (= *k*_*3*_/*k*_*-3*_) and *K*_1_ (= *k*_1_/*k*_-1_) for the Phe(93)E11Tyr(II) mutant are very similar to those for *Ma*Pgb*(II) (Table 2), showing that the conformational switch occurs to a similar extent or with higher efficiency.

Finally, the Phe(145)G8Trp mutant behaves quite similarly to *Ma*Pgb*, showing that mutation of this residue alone is not capable of affecting the overall binding and switching properties of the protein. Indeed, CO appears to behave slightly differently from cyanide; EPR measurements revealed that cyanide binding occurs with a considerable degree of heterogeneity between the high-spin ligand-free ferric form and the ferric cyanide derivative [26]. Remarkably, cyanide could not be trapped in the crystal structure (Fig 9B).

## Conclusion

The integrated use of site-directed mutagenesis, X-ray crystallography, vibrational spectroscopy, and ligand-binding kinetics has allowed the identification of the stereochemical mechanisms that form the basis of CO binding and CO sensing and of the alterations in the conformational state of the heme-binding pocket. The Phe(93)E11 residue acts as a sensor, detecting the presence of the heme-bound ligand through its steric hindrance. Information about the liganded state is transferred to Trp(60)B9, which rearranges its side chain, thus enabling a hydrogen bond to the heme-bound ligand and the closing of tunnel 1. Together with Tyr(61)B10, Trp(60)B9 is responsible for the direct stabilization (*i*.*e*., hydrogen bonding) of the heme-bound ligand, thus facilitating the tuning of ligand affinity. A conformationally heterogeneous distal pocket can be held (at least) partly responsible for the kinetic heterogeneity observed in CO dissociation experiments. Conversely, the structural bases for the heterogeneous ligand-binding kinetics remain unclear. Further experiments are needed, to elucidate the effects exerted by the residues of the proximal heme site and by other residues along the tunnel pathways.

## Supporting Information

S1 Fig Meaningful spectral components (A) and amplitudes (B) as retrieved from the SVD analysis of the time resolved spectra collected after photolysis of the CO adduct of *Ma*Pgb* Tyr(61)B10Ala(II). The first component (black open circles, S_1_ = 27, corr_1_ = 0.99) matches the rebinding kinetics measured at 436 nm (black solid curve), whereas the second component (red open circles, S_2_ = 0.6, corr_2_ = 0.99) shows formation of a reaction intermediate and its decay, similar to the case of *Ma*Pgb*.
(DOCX)

S2 Fig Fitting of the CO rebinding kinetics to the *Ma*Pgb* Phe(145)G8Trp(II) mutant at 100 and 200 μM CO concentration (black open circles) and T = 20°C using the kinetic model in Scheme 1 (yellow curve). (DOCX)

S3 Fig Fitting of the CO rebinding kinetics to *Ma*Pgb* Trp(60)B9Ala(II) at 1 atm CO (black open circles) and T = 20°C using the kinetic model in Scheme 1 (yellow curve). Reaction intermediates are also reported.
(DOCX)

S4 Fig The haem distal site in ferric cyano-met *Ma*Pgb* mutant structures. Residues lining the haem distal pocket are indicated (one letter code) and shown in magenta for the Trp(60)B9Ala mutant (panels A), and in orange for the Tyr(61)B10Ala (panel B). The proximal His(120)F8 residue is also shown. The ferric cyano-met and ligand-free *Ma*Pgb* structures (taken as references) are shown in yellow and grey colors, respectively, and superimposed in panel C (shown from side and top views). The mutated residues have been underlined. H-bonds are shown as dashed lines. Figure adapted from ref. 8 in the main text.
(DOCX)

S1 Table Data collection and refinement statistics for cyanide derivative of ferric *Ma*Pgb* mutants. (DOCX)

S2 Table Positions of the marker bands (ν_4_, ν_3_, and ν_2_), the propionate bending mode (δ (C_β_C_c_C_d_)) and the vinyl bending mode (δ (C_β_C_a_C_b_)) of *Ma*Pgb* mutants in the ferrous forms as observed in the deoxy ferrous RR spectra. (DOCX)

S3 Table Resonance Raman frequencies of the Fe-CO stretching bands (ν_Fe-CO_) in the ferrous CO-bound forms of *Ma*Pgb* mutants. (DOCX)
