# Supplementary material for: Structural Bases for the Regulation of CO Binding in the Archaeal Protoglobin from Methanosarcina acetivorans
Source: PLoS One. 2015 Jun 5;10(6):e0125959. doi: 10.1371/journal.pone.0125959 (PMC4457829; doi:10.1371/journal.pone.0125959)
Supplement: S4 Fig — Residues lining the haem distal pocket are indicated (one letter code) and shown in magenta for the Trp(60)B9Ala mutant (panels A), and in orange for the Tyr(61)B10Ala (panel B). The proximal His(120)F8 residue is also shown. The ferric cyano-met and ligand-free MaPgb* structures (taken as references) are shown in yellow and grey colors, respectively, and superimposed in panel C (shown from side and top views). The mutated residues have been underlined. H-bonds are shown as dashed lines. Figure adapted from ref. 8 in the main text. (DOCX) [file pone.0125959.s004.docx]

###
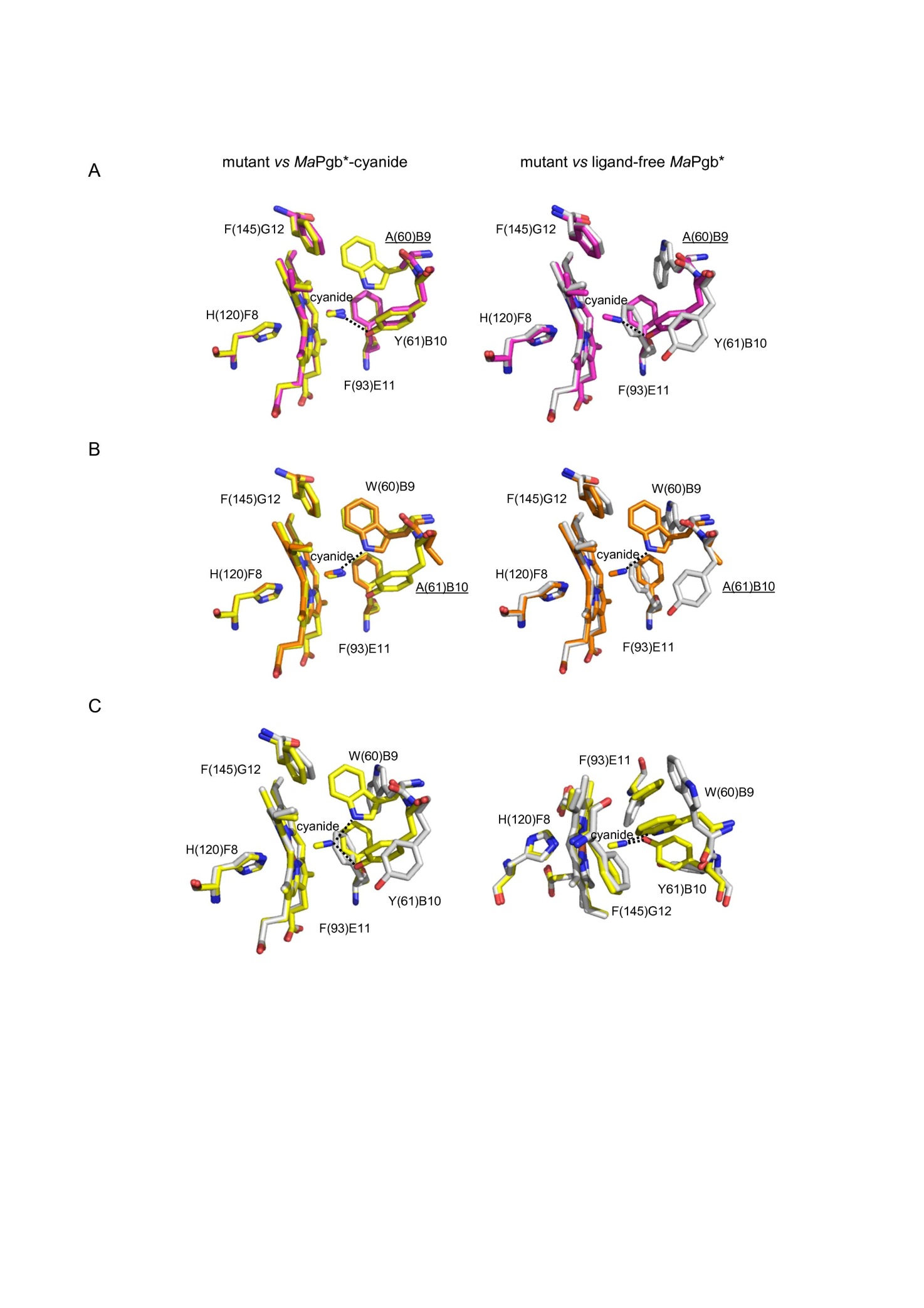


### Figure S4. The haem distal site in ferric cyano-met *Ma*Pgb* mutant structures. Residues lining the haem distal pocket are indicated (one letter code) and shown in magenta for the Trp(60)B9Ala mutant (panels A), and in orange for the Tyr(61)B10Ala (panel B). The proximal His(120)F8 residue is also shown. The ferric cyano-met and ligand-free *Ma*Pgb* structures (taken as references) are shown in yellow and grey colors, respectively, and superimposed in panel C (shown from side and top views). The mutated residues have been underlined. H-bonds are shown as dashed lines. Figure adapted from ref. 8 in the main text.
