# Supplementary material for: Structural Bases for the Regulation of CO Binding in the Archaeal Protoglobin from Methanosarcina acetivorans
Source: PLoS One. 2015 Jun 5;10(6):e0125959. doi: 10.1371/journal.pone.0125959 (PMC4457829; doi:10.1371/journal.pone.0125959)
Supplement: S3 Fig — Reaction intermediates are also reported. (DOCX) [file pone.0125959.s003.docx]

Figure S3. Fitting of the CO rebinding kinetics to *Ma*Pgb* Trp(60)B9Ala(II) at 1 atm CO (black open circles) and T = 20 °C using the kinetic model in Scheme 1 (yellow curve). Reaction intermediates are also reported.
